# Prevalence and incidence of venous leg ulcers—a protocol for a systematic review

**DOI:** 10.1186/s13643-021-01697-3

**Published:** 2021-05-12

**Authors:** S. Probst, C. D. Weller, P. Bobbink, C. Saini, M. Pugliese, Monika Buehrer Skinner, G. Gethin

**Affiliations:** 1 HES-SO University of Applied Sciences and Arts Western Switzerland, School of Health Sciences, Avenue de Champel 47, 1206 Geneva, Switzerland; 2 Department of Epidemiology and Preventive Medicine, School of Public Health and Preventive Medicine, The Alfred Hospital, Monash University, Melbourne, Australia; 3 HES-SO University of Applied Sciences and Arts Western Switzerland, Geneva School of Health Sciences, Geneva, Switzerland; 4 Institute of Epidemiology, Biostatistics and Prevention, Director of Public Health Education Program, University of Zurich, Zurich, Switzerland; 5 School of Nursing and Midwifery, Aras Moyola, NUI Galway, Galway, Ireland

**Keywords:** Venous leg ulcer, Prevalence, Incidence, Epidemiology

## Abstract

**Background:**

Venous leg ulcers (VLUs) are chronic wounds characterized by slow healing and high recurrence. Information on prevalence and incidence is essential for ascertaining the burden of VLU on the health care system and to inform epidemiological research, priority setting, and health care planning. The objective of this protocol is to present a transparent process for how we plan to review the existing international literature on the prevalence and incidence of VLU as well as the characteristics of the population reported within these studies.

**Methods:**

An exploratory search was performed using MEDLINE via PubMed and CINHAL via Ebsco to identify concepts, keywords, MeSH terms, and headings to identify study types looking at data of VLU prevalence and/or incidence and related patient characteristics. The findings of this exploratory search will determine the final search strategy. The titles and abstracts of the identified articles will be screened independently be two authors for relevance. Study which pass the quality assessment will be included. Data extraction will be performed independently by two authors and in accordance with a pre-designed data extraction form. If the data allows, a meta-analysis will be performed otherwise a descriptive summary of the findings will be conducted.

**Discussion:**

The results of this review will contribute to the evidence base on VLU occurrence and may inform the decision making of healthcare professionals, policy-makers, and consumers. It will also inform future research in this area of VLU care.

**Systematic review registration:**

PROSPERO CRD42020205855

## Background

Venous leg ulcers (VLUs) are open lesions of the lower limb and represent between 60 and 80% of all leg ulcerations that occur in the presence of venous disease [1, 2]. Healing rates are protracted with only 60% on average healed by 12 weeks, and once healed, 75% develop a recurrence within 3 weeks [3]. At least 60% of VLUs result in a chronic wound [4]. VLUs are most prevalent among persons of older age with concomitant chronic venous insufficiency. They impact more females than males, those who are obese, immobile, have a congenital absence of veins, or a history of deep vein thrombosis (DVT) or phlebitis [5] resulting in reduced mobility, poor quality of life, and notable financial burden on patients and health care systems [6]. Data from Australia estimate the annual health care costs to VLU treatment of more than AUD$ 3 billion yearly [7] and in the UK at £941 million [8].

Three VLU studies have reported prevalence and incidence of populations in various settings ranging for prevalence from 0.12% [9] to 1.69% [10] and for incidences from 0.3% [11] to 1.33% [10]. This observed variability of occurrence may be in part due to a lack of a clinical registry for VLU [12] and the different methodologies used to collect prevalence and incidence data. As the number of people with VLUs across the globe is expected to rise in the future due to an aging and an increasingly overweight population [2, 13, 14], a systematic collation and review of existing prevalence and incidence studies on VLU will inform decision making, priority setting and health care planning as well as future research.

This protocol for a systematic review will employ strict methodological inclusion and exclusion criteria of published and available literature to identify prevalence and incidence of VLU internationally and will characterize the population as reported in these studies.

The following research questions will be addressed:

What is the prevalence of VLUs for different settings according to internationally published studies?

What is the incidence of VLUs for different settings according to internationally published studies?

What are determinants of VLUs in different settings as reported in these studies?

### Objectives

The objective of this systematic review protocol is to present a transparent process. In particular:

- To systematically search the databases to identify studies in which the prevalence and/or incidence of VLU in any care setting in any country are reported
- To describe information sources of the identified studies reporting prevalence and/or incidence of VLU patients
- To extract and appraise the data from the included studies about prevalence and incidence as well as the population characteristics
- To describe the coding procedures as well as the study quality measures and statistical procedures for the quantitative analysis of data from eligible studies

This systematic review is registered at the International Prospective Register of Systematic reviews (PROSPERO) (CRD42020205855). We will disclose any deviations from this protocol. If so, we will update the PROSPERO record accordingly.

## Methods

We developed this protocol according to the Review and Meta-Analysis–Protocols (PRISMA-P) statement [15, 16].

### Condition

The condition is the VLU as described by authors using the following:

Population: Adults 18 years of age and older with a VLU. The diagnosis of VLU will be as reported within the studies.

Those with any other chronic wound, e.g., arterial ulcer, diabetic foot ulcer, pressure ulcer, burns, or surgical wounds will be excluded.

### Outcome

The primary outcomes will be period prevalence or point prevalence or cumulative incidence or incidence rate of VLU.

### Inclusion and exclusion criteria

We will include intervention studies and observational studies such as cohort studies, case control studies, cross sectional studies. We will exclude editorials, letters, case studies, case series, and animal studies. Studies will be included regardless of language, sample size, or year of publication.

### Information sources

We will search the following electronic databases: Medline (PubMed), Cumulative Index to Nursing and Allied Health Literature (CINAHL) (EBSCO platform), Embase, Scopus, Web of Science, LiSSa (Littérature Scientifique en Santé), Google Scholar, and Cochrane Database of Systematic Reviews.

### Search strategy

The search strategy will be designed and conducted in collaboration with an experienced reference librarian of the HES-University of Applied Sciences and Arts Western Switzerland, Geneva (MP), in consultation with the authors. To guide the electronic literature search strategies, we will use the Peer Review of Electronic Search Strategies (PRESS) 2015 Guideline Statement [17]. To construct a comprehensive set of possible search terms, we will apply controlled vocabulary (e.g., Medical Subject Headings terms) with keywords both in full and in various truncations (see Table 1). Additionally, we will use Boolean operators and proximity operators, including wildcards, AND, OR, parentheses, and quotations for each database. The initial search strategy was designed and piloted on September 2, 2020, and tested for possible study volume on September 7, 2020. We will run the searches firstly with research design filters and then with extensive qualitative filters applied. Table 2 summarizes the search strategy applied for Medline and CINAHL electronic databases.

**Table 1:** Keywords

Concepts	Keywords	MESH (PubMed)	CINAHL headings (CINAHL Complete)
Venous leg ulcer	“venous leg ulcer*”	“varicose ulcer”[MeSH Terms] AND “leg ulcer”[MeSH Terms]	(MH “Venous Ulcer”) AND (MH “Leg Ulcer”)
Prevalence OR incidence	**PubMed** prevalence OR incidence OR occurrence OR epidemiolog*

**Table 2:** Search strategy

Date	Database	Search	Filters or limits	Number of studies
07 September 2020	Medline (PubMed)	(“venous leg ulcer*”[Title/Abstract] OR (“varicose ulcer”[MeSH Terms] AND “leg ulcer”[MeSH Terms])) AND (“prevalence”[Title] OR “incidence”[Title] OR “occurrence”[Title] OR “epidemiolog*”[Title])	None	54
07 September 2020	CINAHL	(TI “venous leg ulcer*” OR AB “venous leg ulcer*” OR ((MH “Venous Ulcer”) AND (MH “Leg ulcer”))) AND (TI prevalence OR TI incidence OR TI occurrence OR TI epidemiolog*)	None	21

## Study records

### Data management

We will import all references into one single EndNote library version X8. Titles will be de-duplicated once entered into EndNote library. We then will export the references from the EndNote Library into the software Rayyan. This software will support the screening process.

### Selection process

Two reviewers (SP, PB) being experts in VLU and conducting reviews in this field will independently screen titles and abstracts for those matching the eligibility criteria. We will retrieve the full-texts of the relevant eligible studies. Two reviewers will independently assess the full texts for study characteristics. The excluded studies will be listed in a table including the reason for exclusion. We will resolve any discrepancies between the reviewers involving a third reviewer. Finally, we will prepare a PRISMA-flowchart to document the final selection process.

### Methodological quality appraisal

Two independent reviewers will conduct a risk of bias assessment; any disagreements will be resolved through discussion or consultation with a third reviewer if needed. To assess the methodological quality of the included studies, the quality appraisal tool for systematic reviews of prevalence data will be used [18]. The quality of evidence will be assessed using the Grading of Recommendations Assessment, Development and Evaluation (GRADE) methods [19].

## Data extraction

Included study data will be extracted and managed independently by two reviewers using an electronic data collection form developed by SP, PB, and MP. The information will include study details (e.g., study ID, author, year, journal), study method (e.g., aims of study, setting, study design, outcomes method of data analysis), and results (e.g., prevalence n/N (%), proportion and 95% confidence intervals (CI), incidence n/N (%), proportion and 95% CI and duration of recruitment or the study). Studies in which wounds of various etiologies are reported will only be included if data specific to VLU can be extracted. If data is unclear or missing, we will contact the authors. We will resolve any disagreements between the reviewers through discussion and if needed by involving a third reviewer.

### Data synthesis

We will summarize the study characteristics and findings descriptively and will present these in tabular format. Prevalence, incidence, and the characteristics of the study population will be summarized and synthesized narratively as well as in tables. If possible, odds ratios (for categorical outcome data) or weighted mean differences (for continuous data) and 95% confidence intervals will be calculated for each included study. To assess the heterogeneity between the studies, we will use the chi-squared test [20]. In the case of a heterogeneity, we will carry out a subgroup analysis (e.g., age, sex, and setting) and univariate meta-regression in order to estimate the effect of study-level covariates on the estimates of prevalence and incidence. If we find a high number of sufficiently homogeneous studies (in terms of study design, population, and outcome characteristics), we will perform a meta-analysis. When pooling proportions for meta-analysis, we will use the Logit transformation to calculate the weighted summary proportion under fixed and random effects models [21]. We will then list the proportions, with their 95% CI, found in the individual studies included in the meta-analysis. We will then present the results graphically in a forest plot. If a meta-analysis is deemed inappropriate, we will present a narrative summary of results as well as in tables/figures, considering the strengths of the studies.

## Discussion

The results of this systematic review will provide key stakeholders with an overview of VLU prevalence, incidence, and VLU determinants. This information will inform healthcare professionals, policy-makers, and consumers in making evidence-based decisions that effectively target and address the VLU burden and will inform future research in this area.

## Data Availability

The datasets used and/or analyzed during the current study are available from the corresponding author on reasonable request.

## Abbreviations

CINAHL: Cumulative Index to Nursing and Allied Health Literature
GRADE: Grading of Recommendations Assessment, Development and Evaluation
LiSSa: Littérature Scientifique en Santé
MeSH: Medical Subject Headings
PRESS: Peer Review of Electronic Search Strategies
PROSPERO: Prospective Register of Systematic reviews
VLU: Venous leg ulcers
